# The Essential Role of Stathmin in Myoblast C2C12 for Vertical Vibration-Induced Myotube Formation

**DOI:** 10.3390/biom11111583

**Published:** 2021-10-26

**Authors:** Yi-Hsiung Lin, Liang-Yin Chou, Hsin-Chiao Chou, Chung-Hwan Chen, Lin Kang, Tsung-Lin Cheng, Chau-Zen Wang

**Affiliations:** 1 Division of Cardiology, Department of Internal Medicine, Kaohsiung Medical University Hospital, Kaohsiung 807, Taiwan; caminolin@gmail.com; 2 Lipid Science and Aging Research Center, Kaohsiung Medical University, Kaohsiung 807, Taiwan; 3 Graduate Institute of Medicine, College of Medicine, Kaohsiung Medical University, Kaohsiung 807, Taiwan; laining59@gmail.com (L.-Y.C.); rna.studio2014@gmail.com (H.-C.C.); hwan@cc.kmu.edu.tw (C.-H.C.); 4 Orthopaedic Research Center, Kaohsiung Medical University, Kaohsiung 807, Taiwan; junglecc@gmail.com; 5 Department of Physiology, College of Medicine, Kaohsiung Medical University, Kaohsiung 807, Taiwan; 6 Division of Adult Reconstruction Surgery, Department of Orthopedics, Kaohsiung Medical University Hospital, Kaohsiung Medical University, Kaohsiung 807, Taiwan; 7 Department of Orthopedics, College of Medicine, Kaohsiung Medical University, Kaohsiung 807, Taiwan; 8 Department of Orthopedics, Kaohsiung Municipal Ta-Tung Hospital, Kaohsiung Medical University, Kaohsiung 801, Taiwan; 9 Regeneration Medicine and Cell Therapy Research Center, Kaohsiung Medical University, Kaohsiung 807, Taiwan; 10 Institute of Medical Science and Technology, National Sun Yat-Sen University, Kaohsiung 804, Taiwan; 11 Department of Obstetrics and Gynecology, National Cheng Kung University Hospital, College of Medicine, National Cheng Kung University, Tainan 701, Taiwan; kanglin@mail.ncku.edu.tw; 12 Department of Medical Research, Kaohsiung Medical University Hospital, Kaohsiung 807, Taiwan; 13 College of Professional Studies, National Pingtung University of Science and Technology, Pingtung 912, Taiwan

**Keywords:** vibration, stathmin, myotube formation, myogenic regulatory factors, PI3K/Akt

## Abstract

Vertical vibration (VV) is a type of whole body vibration, which induces muscle contraction through vibration to improve muscle strength and bone density. However, the mechanism of VV on muscle cell myotube formation is still unclear. In the current study, we aim to clarify the mechanism involved in VV’s stimulation of myotube formation. In order to identify the molecules regulated by VV, we performed proteomics analysis including 2D electrophoresis combined with MALDI-TOF/TOF Mass. Stathmin was identified as a high potential molecule responding to VV stimulation, and we found that under VV stimulation, the expression of stathmin gene and protein increased in a time-dependent manner. In addition, we also confirmed that the increase of stathmin stimulated by VV is mediated through the PI3K/Akt pathway. Furthermore, stathmin siRNA significantly down-regulated the expression of myogenic regulatory factor (MRF) MyoD, decorin, and type I collagen (Col-I), and down-regulated the cellular process regulators such as FGF7, TGFBr1 and PAK3. Taken together, our results confirm that under the stimulation of VV, PI3K/Akt and stathmin would be activated, as well as the up-regulation of MRFs, such as FGF7, TGFBr1 and PAK3 to initiate myogenesis. It also showed that the response of MRF to VV stimulation was significantly related to stathmin expression, which also confirmed the importance of stathmin in the entire myotube formation process. This study may provide evidence of stathmin as a biological indicator of VV to increase muscle strength.

## 1. Introduction

Whole-body vibration (WBV) exercise is a mechanical load that has recently been widely used as a means of physical therapy [1]. Recent studies have demonstrated that the prolonged application of WBV exercise can improve muscle strength and power, body balance and mobility [2,3], especially for athletes or individuals in the absence of gravity, healthy elderly individuals, young adults, and untrained adults to maintain muscle performance [4,5,6]. Vertical vibration (VV) is a type of WBV that can be performed at home, reducing both the therapeutic cost and traveling time of patients [7]. The amplitude and frequency of WBV will vary depending on the treatment, with frequencies and amplitudes of 12 to 45 Hz and 0.35 to 5 mm used in studies. Our previous study indicated that low-magnitude (0.4 mm) VV stimulation at frequencies of 8 to 10 Hz promotes myotube formation by the C2C12 myoblasts. However, the detailed mechanisms of VV-induced myotube formation remain undefined.

During skeletal muscle development, most myoblasts directly differentiate from the somatic mesoderm and secrete myogenic regulatory factors (MRFs) prenatally [8,9]. The myoblasts mature into myocytes and then fuse together as multinucleated myotubes that form the myofibers. MRFs and myocyte enhance factor 2 (MEF2) are essential transcription factors that regulate myogenic differentiation. There are four subfamilies of MRFs, including myogenin, Myf5, MyoD, and myogenic factor 6 (also called MRF4), all of which belong to the basic helix-loop-helix (HLH) protein family [8]. Each MRF has a different function during myogenesis. For example, the overexpression of MyoD results in the conversion of non-muscle cells, such as chondroblasts, fibroblasts and smooth muscle cells, into the skeletal muscle lineage [10]. Our previous studies reported that VV stimulation increases myogenesis-related gene expression and myotube formation by C2C12 myoblasts [11].

Stathmin (also known as stathmin 1, Op18, p18, p19, and metablastin) is a highly-conserved protein in various species and is essential for regulating the cell cycle [12,13], microtubules [14,15] and apoptosis [16,17]. Stathmin is reported to be involved in arsenic trioxide-induced apoptosis in human cervical cancer cell lines [15]. The over-expression of stathmin results in the abnormality of spindle assembling of mitosis, and the inhibition of stathmin leads to the cell cycle arrest in the G2/M phase [18]. However, the role of stathmin in regulating myotube formation has not been revealed yet. Stathmin was reported by L. Casadei et al. in 2009, showing that C2C12 began to differentiate after the differentiation medium stimulation, and stathmin is up-regulated in the cell proliferation initiation, and down-regulated in the subsequent stages. Their results concluded that stathmin plays a key role in cell myoblast proliferation rather than differentiation [19]. In consideration of the high expression level of stathmin at the perinatal stage of myoblast [20], the importance of stathmin toward myotube formation regulated by VV induction should be elucidated.

The PI3K/Akt/mTOR pathway, which is involved in regulating skeletal muscle atrophy, hypertrophy and myotube formation, shows increased activity during muscle hypertrophy and decreased activity during muscle atrophy [21]. Vibration can also induce muscular hypertrophy, which is defined as increased muscle fiber cross-sectional area (CSA) and protein content [6]. An increase in stathmin expression has been correlated with PI3K activity in myeloid leukemia cells [15], breast cancer [22] and endometrial cancers [22,23]. Our previous study showed that inhibition of the PI3K pathway (by using inhibitor LY294002) in C2C12 cells suppresses VV-induced myotube formation [11]. To further investigate the underlying mechanism of VV-enhanced myotube formation, a proteomic analysis was performed to identify novel factors involved in the associated regulatory cascade. In the current study, stathmin siRNA, qRT-PCR and Western blot combined with PI3K/Akt inhibitors were used to elucidate the mechanism of VV-stimulation involved in regulating myotube differentiation. 

## 2. Results

### 2.1. Stathmin Expression Increases in VV-Stimulated C2C12 Cells

**Figure 1:**
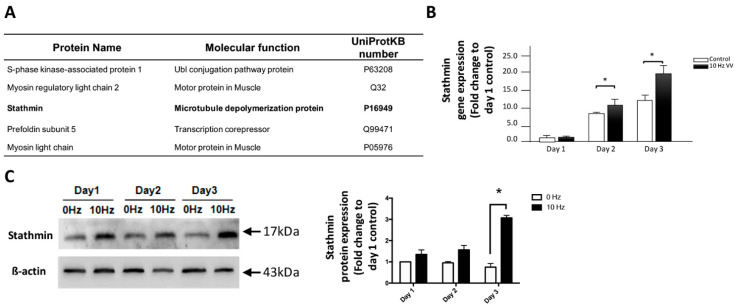
Proteomic analysis of C2C12 myotube formation induced by VV. (**A**) Proteomics analysis identified the list of top five candidate hot spots that VV affected most on C2C12 cells. C2C12 cells treated with 0 or 10 Hz VV stimulation before 2D electronic gel fractionation. (**B**) Stathmin gene expression induced by 10 Hz VV stimulation was further verified by qRT-PCR from days 1 to 3. (**C**) The protein expression and quantitative result of stathmin was determined in a time-dependent manner from days 1 to 3. The data are shown as the means ± SDs of three independent experiments. * *p* < 0.05 is considered significant.

To evaluate the alterations in protein expression after VV treatment, a two-dimensional gel electrophoresis (2-DE) proteomic analysis was conducted. There were several steps leading to hotspot candidates’ identification. First, VV-treated C2C12 cells were harvested and the proteins were extracted for electrophoresis on a 4 to 12% Bis-Tris gradient gel. Second, approximately 270 protein spots with isoelectric points (pIs) ranging from 4.5 to 9.5 and molecular masses ranging from 10 to 240 kDa were visualized by SYPRO Ruby protein gel staining. Third, the majority of proteins located in a pH range of 5 to 8 and had a molecular-mass range of 10 to 50 kDa (Supplementary Figure S1) were targeted. Fourth, among these spots, the top five intense protein spots with high pIs were excised and identified by MALDI-TOF/TOF MS. Fifth, the spots representing proteins, including S-phase kinase-associated protein 1, myosin regulatory light chain 2, stathmin, prefoldin subunit 5, and myosin light chain, were identified (Figure 1A). Among these proteins, stathmin is one of the proteins that has not been confirmed to be related to the differentiation of myoblasts [19], which drew our attention of its role in VV-initiated myotube differentiation. To further investigate the alteration of stathmin stimulated by VV, its gene and protein expression level were evaluated. Using qRT-PCR, we showed that the expression of stathmin was upregulated 10 to 15-fold in a time-dependent manner with VV treatment from day 2 to 3 compared with 0 Hz (control) (Figure 1B). Western blotting results showed that compared with the control at 0 Hz, C212 cells stimulated with 10 Hz VV exhibited significantly upregulated stathmin protein at day 3 (Figure 1C). These results showed that stathmin expression increases in VV-stimulated C2C12 cells.

### 2.2. Stathmin Regulates VV-Stimulated Myotube Formation of C2C12 Cells

**Figure 2:**
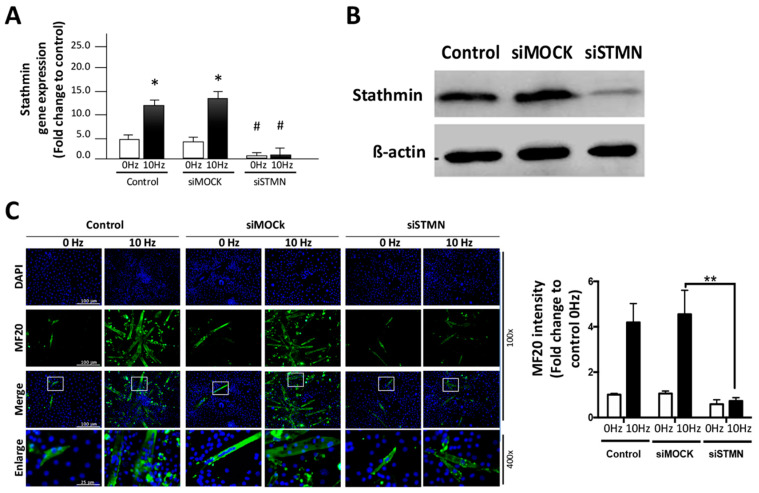
Stathmin siRNA reverses VV-induced C2C12 myotube formation. (**A**) Stathmin siRNA was used to knockdown the upregulated stathmin gene expression observed in C2C12 cells after 10 Hz VV stimulation. siMOCK was used as a vehicle control for stathmin siRNA. (**B**) The protein expression of stathmin was investigated to confirm the efficiency of stathmin siRNA in eliminating protein expression. (**C**) MF20 immunofluorescence and the quantitative results indicated C2C12 myotube formation. C2C12 cells with the indicated VV stimulation were treated with control, siMOCK, and siSTMN to investigate the role of stathmin in VV-induced C2C12 myotube formation. DAPI: nuclear counter stain, MF20: myotube marker. Magnification: 100× and 400× (enlarge). The data are presented as the means ± SDs of three independent experiments. * *p* < 0.05, for each 10 Hz group versus the 0 Hz control groups; # *p* < 0.05, for the stathmin siRNA group versus the siMOCK 10 Hz group. ** *p* < 0.01, for the indicated group comparison.

To further assess the role of stathmin in VV-stimulated myotube formation, gene silencing with stathmin siRNA (siSTMN) was performed. The qRT-PCR results showed that both normal and mock siRNAs (siMOCK)-treated C2C12 cells expressed high levels of stathmin after VV stimulation. However, the siSTMN treatment significantly reduced stathmin expression, even under the 10 Hz VV treatment condition (Figure 2A). The expression of stathmin protein was confirmed by western blot analysis and showed that stathmin protein expression was reduced in C2C12 cells by siSTMN after the 10 Hz VV treatment (Figure 2B). Next, the effect of stathmin siRNA on VV-stimulated myotube formation was assessed. By examining the immunofluorescence of the myotube formation marker MF20 in VV-treated C2C12 cells, we found the strong correlation of stathmin and VV-induced C2C12 myotube formation. Immunofluorescence images and quantitative results showed that 10 Hz VV induced obvious MF20 staining in both the control and siMOCK treatment C2C12 group, compared with that of 0 Hz VV stimulation group. However, the MF20 myotube staining was significantly decreased in the siSTMN treated C2C12, and the quantitative results revealed that knocking down stathmin reduced more than 70% myotube formation induced by VV (Figure 2C). These results indicate that stathmin is critical for the VV-initiated myogenesis.

### 2.3. Stathmin siRNA Significantly Reduces VV-Stimulated MRF Expression

**Figure 3:**
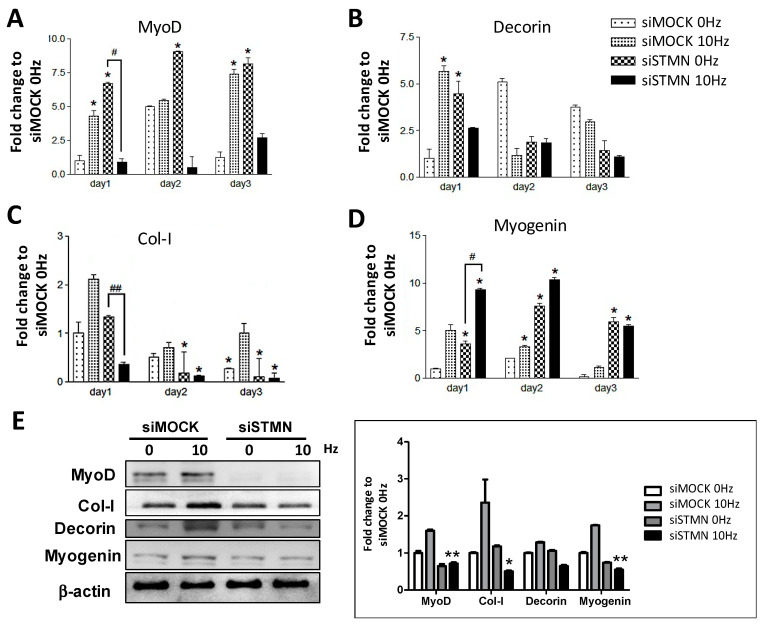
Stathmin siRNA down-regulated VV-stimulated C2C12 MRFs. The effect of stathmin siRNA on the regulation of genes encoding (**A**) MyoD, (**B**) decorin, (**C**) Col-I, and (**D**) myogenin involved in VV-induced C2C12 myotube formation was determined. (**E**) Western blot analysis of MRF Protein expression and related quantitative results of VV and stathmin siRNA regulation. The data are presented as the means ± SDs of three independent experiments. * *p* < 0.05, ** *p* < 0.01 for the siMOCK 10 Hz group versus the siMOCK 0 Hz control group; # *p* < 0.05, ## *p* < 0.01 for the stathmin siRNA 10 Hz group versus the siMOCK 10 Hz group.

Myotube formation by C2C12 cells has been reported to be affected by VV stimulation through the regulation of MRF expression [11]; however, a clear mechanism has yet to be elucidated. In the current study, the expression of myotube formation-related regulatory genes, including MyoD, decorin, collagen type I (Col-I), and myogenin were investigated by using qRT-PCR. As shown in Figure 3A, MyoD was upregulated by VV stimulation but significantly downregulated in VV-treated stathmin-knockdown C2C12 cells. Compared to the 0 Hz control group, VV-treated C2C12 cells expressed a high level of decorin, which was significantly decreased by stathmin silencing one day after induction (Figure 3B). The synthesis of the extracellular matrix component Col-I in response to VV stimulation was also investigated. As shown in Figure 3C, Col-I was upregulated by the VV treatment, and siSTMN significantly reduced the Col-I expression induced by the 10 Hz VV treatment. Similar results were observed on days 2 and 3 for the VV-stimulated Col-I upregulation and suppression by stathmin siRNA treatment. These results suggest that VV-stimulated C2C12 myotube formation occurs through a stathmin-mediated pathway to regulate MyoD, decorin and Col-I transcription. Interestingly, Figure 3D showed that VV-stimulated C2C12 cells increased myogenin transcription, whereas stathmin siRNA further increased myogenin transcription; this suggests that the VV-initiated myogenin up-regulation might be through a stathmin-independent pathway. Furthermore, the protein expression of the myogenesis-related protein under VV and siRNA of stathmin treatment had been investigated through Western blot to better interpret the role of stathmin in VV induced myogenesis. The results showed that knockdown stathmin deregulated the expression of MyoD, decorin and Col-I, which suggests the regulatory effect of stathmin toward those MRFs (Figure 3E).

### 2.4. PI3K/Akt Pathway Are Correlated Regulators of Stathmin in VV-Stimulated C2C12 cElls

**Figure 4:**
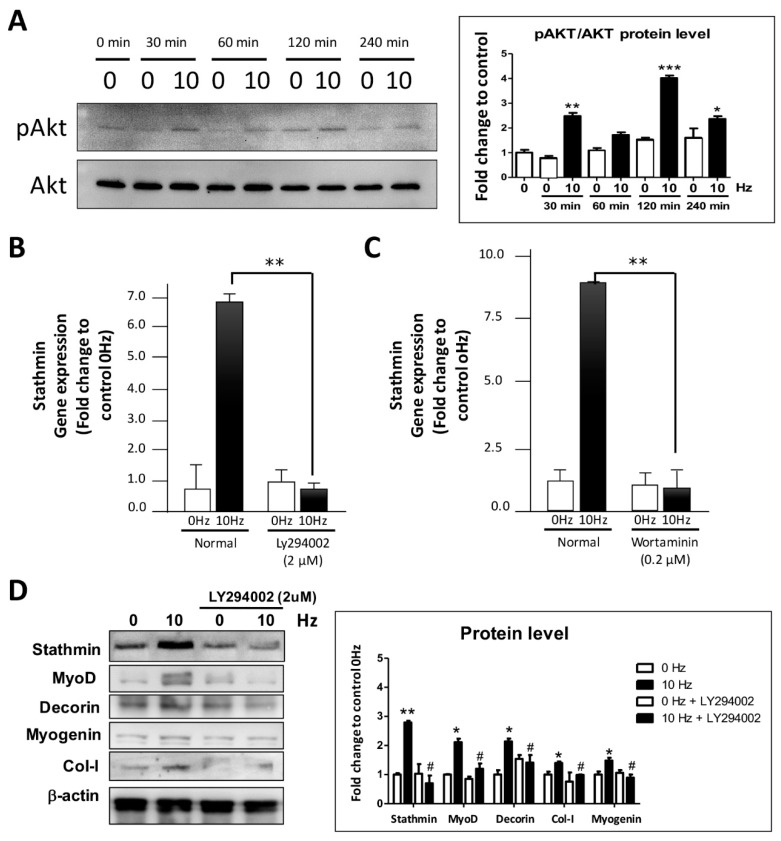
VV-induced stathmin upregulation and myotube formation through the PI3K/Akt pathway. (**A**) The level of Akt phosphorylation in C2C12 cells induced by 10 Hz VV in a time-dependent manner from 30 to 240 min was investigated by Western blotting. The phosphorylation of Ser473 of Akt was determined and compared with total Akt expression. PI3K-specific inhibitors, including (**B**) Ly294002 (2 µM) and (**C**) wortmannin (0.2 µM), were used to treat 10 Hz VV-stimulated C2C12 cells to elucidate the regulatory effect of the PI3K/Akt pathway on stathmin gene expression. (**D**) Protein modification of PI3K/Akt-associated molecules including stathmin, and related MRFs regulation under the condition of VV stimulation and PI3K/Akt inhibitor Ly294002 (2 µM) treatment. * *p* < 0.05, ** *p* < 0.01, *** *p* < 0.001 were considered significant compared with individaul 0 Hz control. # *p* < 0.05 compared with the individual 10 Hz only group.

The PI3K/Akt pathway has been reported to be highly associated with myotube formation [24]; thus, the phosphorylation of Akt was investigated by Western blot analysis. The results showed that Akt phosphorylation increased in a time-dependent manner from 30 min to 240 min after VV treatment. These results indicated that VV stimulates myotube formation via the PI3K/Akt-associated pathway (Figure 4A). To characterize the association of stathmin in Akt-mediated myotube formation, the PI3K/Akt-specific inhibitors Ly294002 (2 µM) and wortmannin (0.2 µM) were used. The qRT-PCR results showed that compared with the 0 Hz control, 10 Hz VV stimulation upregulated stathmin gene expression by 8-fold; however, both Ly294002 (Figure 4B) and wortmannin (Figure 4C) significantly suppressed the stathmin expression induced by 10 Hz VV treatment. These results indicated that VV stimulates stathmin up-regulation via a PI3K/Akt-dependent pathway. In addition, we also investigated the alteration of MRF protein to further reveal the role of PI3K/Akt in regulating VV-induced stathmin and MRFs activation in the process of myogenesis. The results clearly indicate that specific inhibitor Ly294002 inhibited the activation of PI3K/Akt, caused the suppression of stathmin induced by VV, and led to the myogenesis-related molecule reduction (Figure 4D).

### 2.5. TGFBr1, FGF7 and PAK3 Are Involved in VV-Induced Myotube Formation and Are Affected by Stathmin Absence

**Figure 5:**
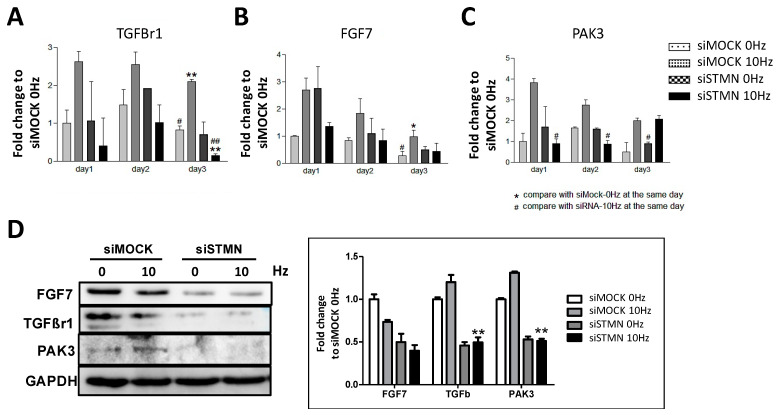
Stathmin correlated regulator activation under the stimulation of VV. Gene expression of (**A**) TGFBr1, (**B**) FGF7, and (**C**) PAK3 regulation under the stimulation of VV and stathmin siRNA for 24–72 h. (**D**) The protein expression of FGF7, TGFBr1 and PAK3 expression responding to the treatment of VV and stathmin siRNA for 24 h. * *p* < 0.05, ** *p* < 0.01 for the siMOCK 10 Hz group versus the siMOCK 0 Hz control group; # *p* < 0.05, ## *p* < 0.01 for the stathmin siRNA 10 Hz group versus the siMOCK 10 Hz group.

To investigate the regulation of genes involved in stathmin-mediated VV-stimulation myotube formation, genes of interests were characterized. The qRT-PCR results showed that the VV-treated C2C12 cells had increased TGFBr1 transcription by 2.5-fold in a time-dependent manner from days 1 to 3. However, inhibition of stathmin significantly reduced VV-stimulated TGFBr1 transcription (Figure 5A). In addition, the qRT-PCR results showed a 2.8-fold increase in FGF7 expression in VV-stimulated C2C12 cells but was decreased to nearly normal expression levels by stathmin knockdown from days 1 to 3 (Figure 5B). Moreover, PAK3 was observed to be upregulated by 3.9-fold from days 1 to 3 after VV treatment; however, stathmin siRNA reduced PAK3 expression only on days 1 and 2 and not day 3. These results indicated that stathmin-regulated transcription of PAK3 occurs during the early stages of VV-stimulated myotube formation (Figure 5C). Likewise, the protein expression of those regulators were investigated by Western blot analysis. The results showed that both TGFBr1 and PAK3 were up-regulated after VV stimulation, and decreased by siSTMN treatment. However, FGF7 protein did not respond to the VV stimulation, but was still reduced by stathmin siRNA treatment. The discrepancy of FGF7 mRNA and protein responding to VV stimulation could be because of the mRNA-to-protein translation buffering. Nevertheless, the results indicate that TGFBr1, FGF7 and PAK3 are correlated regulators of stathmin, and are involved in VV-enhanced myotube formation.

## 3. Discussion

VV is a mechanical load that is widely used to improve muscle strength and joint flexibility during patient rehabilitation. There are increasing reports on how VV activates mechanical stress-responsive cells, both in tendon- and muscle-associated cells. Our previous studies have revealed the regulatory effect of VV in manipulating tenogenic gene expression to improve tendon strength and stiffness, and its beneficial effects on muscle cell differentiation have been previously demonstrated [11,25]. The current study was an extension of the previous findings to explore the mechanism and the potential of stathmin as an emerging biomarker for myogenesis. The effects of vibration therapy on muscle development in the human body have been clinically demonstrated [26], and these exercises have also been shown to have profound benefits in preventing muscle atrophy for improving muscle strength [7]. Multiple signaling events, including gene transcription, translation and cell behavior alteration have been reported to be involved in the response to vibration therapy through deformation of the plasma membrane. In this study, through proteomic analysis using 2D electronic gel separation and mass spectrometry, stathmin was identified as a novel regulator that is significantly involved in myotube formation. We found that the mechanism by which VV induced C2C12 cells to form myotubes had three different manners: (1) the activation of the PI3K/Akt pathway by increasing its phosphorylation status; (2) the upregulation of stathmin; and (3) the activation of MRFs, FGF7, TGFBr1 and PAK3 in response to VV-stimulated myotube formation. Stathmin plays an important role in the whole VV-initiated myotube formation. Our results indicate that if stathmin is absent, these related regulators would not express correctly, and eventually affect myogenesis.

Accumulated reports indicate that the PI3K/Akt pathway is significantly involved in the development and hypertrophy of skeletal muscle by regulating myotube formation. [27,28]. However, to date, few markers have been identified that are involved in the PI3K/Akt regulation of MRFs. In this study, we provided evidence on stathmin as a potential regulator of PI3K/Akt signaling and MRF expression, including MyoD, decorin, and Col-I, to promote C2C12 myotube formation under VV stimulation. However, we failed to observe the phenomenon of stathmin regulating myogenin. Myogenin was increased by VV-stimulation, and was not decreased by stathmin siRNA. We speculate that the possible reason may be that myoblasts are more related to myoblast irreversible cell cycle arrest, and this pathway has been reported to be regulated by TAp63γ instead of stathmin. Therefore, the irrelevance of myogenin and stathmin could be understandable [29].

In addition to the expression of regulatory factors related to myotube formation, we also studied the regulatory molecules involved in VV effect, including TGFBr1, FGF7 and PAK3, and discussed the regulatory role of stathmin on these molecules in the process of VV-induced C2C12 differentiation. Accordingly, TGFBr1 has been shown to regulate C2C12 myoblast proliferation and differentiation by triggering the Smad3 signaling pathway [30,31]. In addition, TGFBs and TGFBRs are reported to be highly associated with myoblast differentiation (including fusion and myogenesis) and as the regulator of MRFs [32,33,34]. It has been reported that FGF7 is up-regulated in differentiated myofibers [35,36]. FGF6 is the most well-known factor related to muscle differentiation in the FGF family. Our results also preliminarily confirmed the increase of FGF7 in VV-initiated myoblast differentiation. PAK3 is an important promoter regulator of the cell cycle for cell proliferation [37,38,39]. Joseph et al., in 2019, concluded that PAK1 and 2 are the components of the promyogenic Ncad/Cdo/Cdc42 signaling pathway for positive myogenic differentiation. PAK3 is similar to PAK2 and is predicted to play a similar role in promoting myogenic differentiation [39]. However, apart from TGFBr1, there is no direct evidence to confirm the regulatory effect between FGF7/PAK3 and MRF. This also leads us to further study the interaction and regulation between each other. 

Besides, there is also no evidence confirmed for the direct interaction of stathmin onto the three factors gene activation zone, but lots of studies indicate that a stathmin-interacting transcription factor, STAT3, is required for the activation of TGFBr1 [40,41]. The absence of stathmin would cause the STAT3 complex to be unstable, and eventually lead to the down-regulation of TGFBr1, PAK3 and FGF7. Therefore, stathmin may play an important role in maintaining the STAT3 complex as a transcription factor in response to VV stimulation and phosphorylation of related kinases.

Although the impact of VV on the disease improvement remains to be supported by more evidence, there have been many studies and reports regarding the effect of vibration therapy to support its efficacy. Elfering, in 2011, proved that low-frequency whole-body vibration therapy provided significant help to patients’ pain control and balance function [42]; and Van Nes’ study, in 2004, suggests the positive effect of systemic vibration therapy in improving standing balance ability and the center of gravity shift speed of stroke patients without side effects, such as dizziness and falls [43]. Besides, it was shown that a single vibration stimulation enhanced muscle strength muscular power of the lower extremity [44,45]. Stathmin is a detectable molecule in blood serum. When VV is applied to improve muscle strength of stroke patients, checking blood stathmin concentration as a biological indicator could be a possible strategy.

## 4. Materials and Methods

### 4.1. Culture

The mouse skeletal myoblast line C2C12 (CRL-1772) (American Type Culture Collection; ATCC, Manassas, VA, USA) was grown in high-glucose Dulbecco’s modified Eagle’s medium (DMEM) supplemented with 10% fetal bovine serum (FBS), 100 units/ml penicillin, and 100 µg/ml antimycoplasma. The myoblasts were maintained in a 5% CO_2_ atmosphere at 37 °C.

### 4.2. Vertical Vibration (VV) and Inhibitor Treatment

The vibration stimulation treatment was performed with the vertical platform (BodyGreen, Taiwan) using a 10 Hz frequency and 0.4 mm amplitude at 10 min/per day. LY294002 (PI-103, Selleckchem, Houston, TX, USA) and wortmannin (KY12420, Sigma Aldrich Inc., Taiwan) are phosphoinositide 3-kinase PI3K/Akt pathway inhibitors. The inhibitors were dissolved in dimethyl sulfoxide (DMSO) at concentrations of 1 mM (wortmannin) and 20 mM (LY294002), and subsequently stored at −80 °C. C2C12 cells were seeded at a high density of 2 × 10^5^ cells/cm^2^ into a 6 cm dish. The cells were starved in DMEM supplemented with 0.5% FBS for 16 h. Subsequently, the cells were pretreated with the PI3K inhibitor LY294002 at 20 µM, wortmannin at 100 µM, or 0.01% DMSM (vehicle control) for 1 h, which was followed by VV for 3 days, and then cultured for an additional 3 days (total of 6 days). Myotube formation was analyzed by immunofluorescence or Western blot analyses.

### 4.3. Two-Dimensional Gel Electrophoresis (2-DE)

C2C12 cells were seeded at a high density into a 6 cm dish and were treated with vibrational stimulation (10 Hz) or were left untreated (0 Hz) for a 3 day stimulation, which was followed by culturing for an additional 3 days. Protein lysis was performed in a sample buffer that contained 8 M urea, 100 mM DTT, 4% CHAPS, 0.2% carrier ampholytes, 40 mM Tris and 0.002% bromophenol blue dye dissolved in 50 mL double-distilled water (ddH2O). Subsequently, 2-DE was performed using a ZoomTM IPGRunnerTM mini-cell kit (ZM0001, Thermo Fisher Scientific Inc., Waltham, MA, USA) according to the manufacturer’s protocol, which was followed by liquid chromatography-tandem mass spectrometry (LC-MS) proteomic analysis (LC-MS Waters ZQ 4000 single Quad Mass Spectrometer; MYCO Instrumentation, Inc, Bonney Lake, WA, USA). Three independent replicate experiments with matched control samples were performed to assess the significance of the data.

### 4.4. Myotube Formation Immunofluorescence

The experimental protocol followed the previous publication [11]. In brief, C2C12 cells were fixed with 4% paraformaldehyde for 20 min, and then washed with PBS 3 times. After punching with 0.5% Triton X-100, they were rinsed with PBS again. They were stained with anti-myosin antibody (MF-20) for 1 h at room temperature, and then washed 3 times in PBS. Secondary antibody labeled with Alexa Fluor 488 (Molecular Probes, Eugene, OR, USA) was added at room temperature for 1 hour, and then DAPI was used for counter stain. Representative images were captured and scored by a fluorescence microscope, including the number and length of myotubes, the occupied area of myotubes relative to the total area, as well as the fusion index (%) within multinucleated myotubes. The parallel side by side specimens sample was used as a negative control.

### 4.5. Stathmin Knockdown Using Specific Stathmin siRNA in C2C12 Myoblasts

To knockdown stathmin in C2C12 myoblasts, the cells were transfected with stathmin siRNA (siSTMN) (SC-36128; Santa Cruz Biotechnology, Dallas, TX, USA) and mock siRNAs (siMOCK), with scrambled sequences corresponding to stathmin (SC-37007; Santa Cruz Biotechnology, Dallas, TX, USA) as controls. C2C12 cells were seeded at a high density of 2 × 10^5^ cells/cm^2^ into a 6 cm dish and starved for 16 h. Subsequently, the siRNA was transfected into C2C12 cells using Lipofectamine 2000 (Invitrogen, Waltham, MA, USA) following the manufacturer’s protocol. Subsequently, stathmin expression was assessed by qRT-PCR and Western blotting.

### 4.6. Immunofluorescence

C2C12 cells were seeded at 1 × 10^4^ cells/cm^2^ into culture chambers and pretreated with LY294002. After VV stimulation for 3 days and culturing for an additional 6 days (total of 9 days), the cells were washed twice with 1× PBS and then incubated with 4% formaldehyde for 20 min. The cells were then washed 3 times with 1× PBS for 5 min each and then blocked with 5% bovine serum albumin in 1× PBS for 1 h. Subsequently, the cells were incubated with an anti-Myosin 4 (MF-20) primary antibody (1:200; 14-6503-80, Thermo Fisher Scientific Inc., Waltham, MA, USA) at 4 °C overnight. The cells were then washed with 1× PBS containing 0.05% Tween-20 (PBST) for 10 min and then incubated with mouse IgG (H+L) highly cross-adsorbed secondary antibody (A32723, Thermo Fisher Scientific Inc., Waltham, MA, USA). Subsequently, the cells were washed 3 times with PBST for 10 min each, counterstained with Gold Antifade Reagent containing DAPI (1:1000; #8961, Thermo Fisher Scientific Inc., Waltham, MA, USA), mounted with mounting media (P36930, Thermo Fisher Scientific Inc., Waltham, MA, USA) and observed on a Zeiss AxioPlan2 fluorescence microscope (Zeiss Microsystems, Marzhauser, Germany).

### 4.7. Western Blot Analysis

C2C12 cells were seeded at 2 × 10^5^ cells/cm^2^ into a 6 cm dish, and cell lysates were collected after VV stimulation from days 1 to 3. RIPA Lysis and Extraction Buffer (89900, Thermo Fisher Scientific Inc., Waltham, MA, USA) containing protease and phosphatase inhibitor (1:100; A32961, Thermo Fisher Scientific Inc., Waltham, MA, USA) was used to extract the cell lysate. Western blot analysis was performed with primary antibodies against stathmin (1:1000), decorin (1:1000), Col-I (1:500), FGF7 (1:1000), TGFBr1 (1:500) and PAK3 (1:500) (Abcam Inc., Cambridge, MA, USA), pAKT (1:500), AKT (1:2000), MyoD (1:1000) and myogenin (1:500) (Cell Signaling Inc., Danvers, MA, USA), β-actin (1:10,000) (Arigo Biolaboratories Corp., TE Huissen, Netherlands), which were incubated with the membranes at 4 °C overnight. After being washed 3 times for 15 min each, the membranes were incubated with an HRP-conjugated goat antibody anti-mouse or anti-rabbit IgG secondary antibody (Arigo Biolaboratories Corp., TE Huissen, Netherlands) for 1 h at RT. The proteins were detected using an ECL substrate kit (Abcam Inc., Cambridge, MA, USA). Band intensity was quantified by using ImageJ software (National Institutes of Health, Bethesda, MD, USA). Intensity data were normalized to GAPDH and indicated controls for the target protein fold changes calculation. 

### 4.8. Quantitative Real-Time PCR (qRT-PCR) Analysis

RNA was extracted using the PureLink RNA Mini Kit (89900, Thermo Fisher Scientific Inc., Waltham, MA, USA), and 2 µg of mRNA was reverse-transcribed into cDNA using the SuperScript II First-Strand Synthesis System (Invitrogen, Carlsbad, CA, USA) with a Perkin-Elmer Gene Amp 9700 PCR system. The reaction buffer (Toyobo Inc., Osaka, Japan) with specific primers (Table 1) was mixed with the cDNA, and PCR was performed using a T100TM Thermal Cycler (Bio-Rad Laboratories Inc., Hercules, MA, USA). The calculation of gene expression ΔCt and ΔΔCt values is normalized by using the Ct value of the housekeeping gene GAPDH and the indicated control for the relative quantification of target gene expression fold change.

### 4.9. Statistical Analysis

All data were obtained from at least three independent experiments and were expressed as the means ± standard deviation (SD) of the combined data from each experimental replicate. One-way analysis of variance was used to assess differences between groups, and multiple comparisons were performed using Scheffe’s method. *p* < 0.05 was considered significant.

## 5. Conclusions

**Figure 6:**
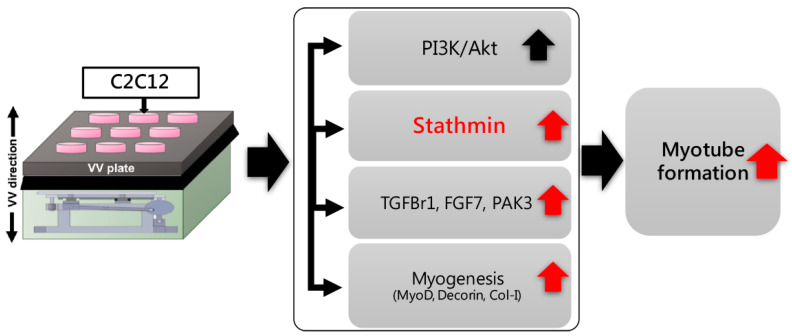
Proposed mechanism whereby VV stimulated myoblast C2C12 myotube formation through a stathmin-dependent myotube formation. Stathmin plays a crucial role in regulating MRFs MyoD, decroin and Col-I, as well as the cellular functional molecules TGFBr1, PAK3 and FGF7 expression, during myogenesis.

Taken together, the results of this study provided evidence that VV enhanced myotube formation through the PI3K/Akt/stathmin pathway, and stathmin was essential for MRFs such as MyoD, decorin, and Col-I expression during myogenesis. Moreover, it was also found that TGFBr1, PAK3 and FGF7 were positively regulated by stathmin and participated in the mechanisms of VV-induced myogenesis (Figure 6). These findings support the practical value of VV in the treatment of diseases characterized by improved myotube formation. In addition, it also proved that stathmin is essential for VV-induced myotube formation and has the potential as a biomarker of VV to increase muscle strength.

## Figures and Tables

**Table 1:** Primer list.

Gene Symbol	Forward	Reverse
Stathinin	5′-CCAGGCTTTTGAGCTGATTC-3′	5′-GCGTCTTTCTTCTGCAGCTT-3′
MyoD	5′-GCTTCTATCGCCGCCACTCC-3′	5′-CGCACATGCTCATCCTCACG-3′
Decorin	5′-ACAGCATCACCGTTATGGAGAATG-3′	5′-TCACAGCCGAGTAGGAAGCC-3′
Collagen type I	3′-TCAGAGGCGAAGGCAACAGTC-3′	3′-GCAGGCGGGAGGTCTTGG-3′
Myogenin	5′-GCATGCAAGGTGTGTAAGAG-3′	5′-GCGCAGGATCTCCACTTTAG-3′
p53	5′- GATGACTGCCATGGAGGAGT -3′	5′- CTCGGGTGGCTCATAAGGTA -3′
GAPDH	5′-ATTGTGGAAGGGCTCATGACC-3′	5′-ATGCAGGGATGATGTTCTGGG-3′
FGF7	5′-GACAAACGAGGCAAAGTGAAAGG-3′	5′-TGCCACAGTCCTGATTTCCA-3′
TGFBrl	5′-CCGCAACAACGCCATCTATG-3′	5′-CCCGAATGTCTGACGTATTGAAG-3′
PAK3	5′-AAATTGGTCAAGGGGCATCAG-3′	5′-ACCCATAGTTCATCACCCACC-3′

## Data Availability

Not applicable.

## Supplementary Materials

The following are available online at https://www.mdpi.com/article/10.3390/biom11111583/s1, Figure S1: Two-dimensional gel electrophoresis (2-DE) proteomic analysis of C2C12 cell protein regulation initiated by VV.

## Abbreviations

Cell Division Cycle 42 (Cdc42); Cross-sectional area (CSA); Fibroblast Growth Factor 6 (FGF6); Fibroblast Growth Factor 7 (FGF7); Myogenic regulatory factor (MRF); Myogenic differentiation factor (MyoD); Mechanistic target of rapamycin (mTOR); Mock siRNA (siMOCK); Myocyte Enhancer Factor 2 (MEF2); Myogenic Factor 5 (Myf5); N-cadherin (Ncad); Phosphoinositide 3-kinases (PI3K); Protein kinase B (Akt); P21 Activated Kinase 3 (PAK3); Stathmin siRNA (siSTMN); SMAD Family Member 3 (Smad3); Signal Transducer and Activator of Transcription 3 (STAT3); Transforming Growth Factor Beta Receptor 1 (TGFBr1); Transactivation p63 isoform γ (TAp63γ); Type I collagen (Col-I); Vertical vibration (VV); Whole-body vibration (WBV).
