# Supplementary material for: The Essential Role of Stathmin in Myoblast C2C12 for Vertical Vibration-Induced Myotube Formation
Source: Biomolecules. 2021 Oct 26;11(11):1583. doi: 10.3390/biom11111583 (PMC8615486; doi:10.3390/biom11111583)
Supplement: Supplementary file 1 [file biomolecules-11-01583-s001.zip › biomolecules-1358477-supplementary.pdf]

Supplementary figure S1.

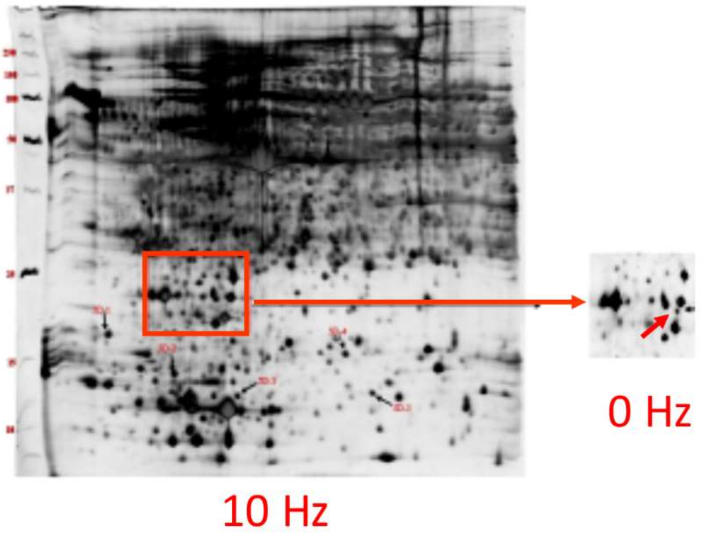

Figure S1. Two-dimensional gel electrophoresis (2-DE) proteomic analysis of C2C12 cell protein regulation initiated by VV. 2-DE was used to compare the changes in protein expression of C2C12 cell differentiation induced by 10 Hz VV. Subsequently, the top 5 candidate protein spots with the most significant differences were identified by MALDI-TOF/TOF MS.
